# Detection of Begomovirus in chilli and tomato plants using functionalized gold nanoparticles

**DOI:** 10.1038/s41598-021-93615-9

**Published:** 2021-07-09

**Authors:** R. Lavanya, V. Arun

**Affiliations:** Department of Biotechnology, Sri Ramachandra Institute of Higher Education and Research, Chennai, Tamil Nadu India

**Keywords:** Biotechnology, Plant sciences

## Abstract

Begomoviruses are a major class of Geminiviruses that affects most dicotyledonous plants and causes heavy economic losses to farmers. Early detection of begomovirus is essential to control the spread of the disease and prevent loss. Many available detection methods like ELISA, immunosorbent electron microscopy, PCR or qPCR require expertise in handling sophisticated instruments, complex data interpretation and costlier chemicals, enzymes or antibodies. Hence there is a need for a simpler detection method, here we report the development of a visual detection method based on functionalized gold nanoparticles (AuNP assay). The assay was able to detect up to 500 ag/µl of begomoviral DNA (pTZCCPp3, a clone carrying partial coat protein gene) suspended in MilliQ water. Screening of chilli plants for begomoviral infection by PCR (Deng primers) and AuNP assay showed that AuNP assay (77.7%) was better than PCR (49.4%). The AuNP assay with clccpi1 probe was able to detect begomoviral infection in chilli, tomato, common bean, green gram and black gram plants which proved the utility and versatility of the AuNP assay. The specificity of the assay was demonstrated by testing with total DNA from different plants that are not affected by begomoviruses.

## Introduction

Plant pathogens and pests are some of the major factors that limit crop productivity. Infection could be detected easily by visual inspection if the characteristic symptoms of the disease are clearly visible. However many factors play a major role in identification of infection like viral strain, cultivar, time of infection and other environmental factors^1^. Geminiviridae and Potyviridae are the two largest families that constitute important plant viruses, which are highly devastating phytopathogens worldwide^2,3^. The family Geminiviridae was established in 1978^4^ and consists of 9 genera: *Becurtovirus, Capulavirus, Curtovirus, Eragrovirus, Grablovirus, Mastrevirus, Topocuvirus, Turncurtovirus* [Monopartite genome] and *Begomovirus* [Bipartite genome]^5–7^. The genetic material of geminiviruses is ssDNA and has either a monopartite (DNA-A) or bipartite (DNA-A and DNA-B) genome. Of the many geminiviruses infecting economically important crops, begomoviruses are one of the most destructive viruses and are transmitted by whitefly vector *Bemisia tabaci*^8–10^.

*Capsicum annuum* (Chilli) and *Solanum lycopersicum* (Tomato) plants belong to the family Solanaceae and are grown in tropical countries. It is a family of flowering plants, consisting of many important crops like potatoes, tomatoes, peppers, tobacco, petunia and other crops of regional significance^11,12^. Plants belonging to the Solanaceae family are heavily affected by viruses and begomoviruses are the major pathogen^8,13–16^. Begomovirus symptoms in plants range from symptomless to different degrees of stunting and curling, distortion, mosaic, mottling, vein yellowing of leaves, flower abortion, small and unmarketable fruits^17^. India is one of the top producers of chilli and tomato in the world and are cultivated across the country throughout the year^18^. Begomoviral infection has been reported in different states of India in chilli and tomato plants and were shown to cause severe losses^19^. Begomovirus infecting pepper (chilli) and tomato plants have narrow host ranges and were shown to infect plants of other families, e.g., *Fabaceae* [common bean and soybean (*Glycine max*)] and *Cucurbitaceae*^20,21^.

One of the basic requirements for the elimination and spread of viruses is detection of the presence of viruses at the earliest possible stage in plants^22^. Many methods are available for the detection of plant viruses which could be classified into protein-based methods (immunotechniques) or nucleic acid-based methods (molecular methods). The various immunotechniques include enzyme linked immunosorbent assay (ELISA)^23–25^, immunoblotting^26^, immunosorbent electron microscopy^27,28^ and precipitin tests^29^. These methods rely on specific interaction of antibody (monoclonal or polyclonal) with viral antigens (coat protein) and hence are prone to false-negative results at low viral titers. The limitations of these methods also include cross-reactivity with similar antigens resulting in false-positive results. The production of antibodies is tedious, expensive and requires expertise.

Molecular methods like PCR^30–33^, Nucleic Acid Spot Hybridization (NASH) assay^34–40^ depend on viral nucleic acids and viral specific primers or probes. PCR is the most commonly used detection technique for begomoviruses and primers were primarily designed on coat protein and replicase genes^41^. Among the viral proteins, coat protein has been shown to be important for the infection and propagation of the virus. This gene was reported to be conserved among begomoviruses and many primers designed in this region were utilized for PCR based detection of begomovirus^42^. The major advantages of molecular techniques are specificity, sensitivity, faster detection and multiplexing^43,44^. Though most molecular techniques are robust, there are drawbacks like non-specific amplification, inhibition of polymerase activity due to the presence of phenolics, polysaccharides in the nucleic acids^45^.

Simple and convenient technologies for the identification of chemical and biological species are of great significance in environmental monitoring, public health and disease diagnosis^46,47^. Here we report the development of functionalized gold nanoparticles (AuNPs) based visual detection method that rely on the hybridization principle. Gold nanoparticles (AuNPs) have been routinely used for the development of visual detection methods for many pathogens due to their unique colour properties and tunable localized surface plasmon resonance (SPR)^48^. Surface plasmon resonance has been shown to be dependent on NP (nanoparticle) composition, size and shape^49,50^.

**Figure 1:**
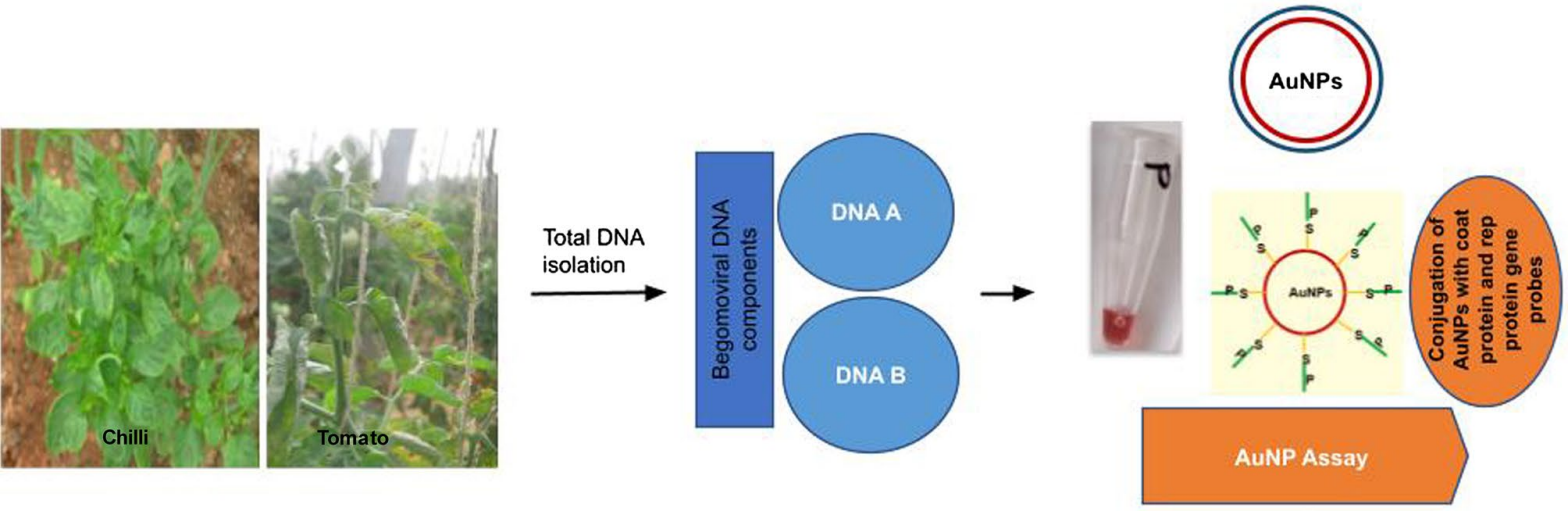
Schematic representation of the AuNP assay proposed in this work. Total DNA would be isolated from plant samples and screened for the presence of begomoviruses using the functionalized AuNPs.

Detection methods based on functionalized nanoparticles were found to be highly sensitive with faster turnover than the other methods like PCR, ELISA. Early detection of plant pathogens is critical for the management of infections in plants as it would result in better segregation of healthy plants and infected plants at the earliest, thus preventing the spread of disease in a farm or field^51^. Recently, a visual DNA method using integrated Recombinase Polymerase amplification (RPA) and a AuNP probe has been reported for detection of tomato yellow leaf curl virus and was found to be highly sensitive and stable^52^. In our lab, we had developed a method for detection of Banana Bunchy Top Virus (BBTV) using functionalized AuNPs^53^. In this work, we demonstrated the applicability of functionalized AuNPs in the detection of begomoviruses in plants and an Indian patent has been filed (Application No: 201941007582) (Fig. 1).

## Results

### DNA isolation from chilli and tomato leaves

A total of 99 chilli samples and 79 tomato samples were used in this study and total DNA was isolated using the Dellaporta method. The total DNA obtained from chilli and tomato plants were analyzed by agarose gel electrophoresis and Nanodrop. The DNA obtained from tomato samples had brownish tinge and were viscous, whereas DNA from chilli samples had aqueous consistency and were transparent. Total DNA from other plants (*Phaseolus vulgaris*, *Vigna radiata*, *Vigna mungo*, *Indigofera aspalathoides*, *Plectranthus amboinicus* and *Catharanthus roseus*) used in this study also had good quality.

### Screening of chilli and tomato plants by PCR for begomovirus infection

**Figure 2:**
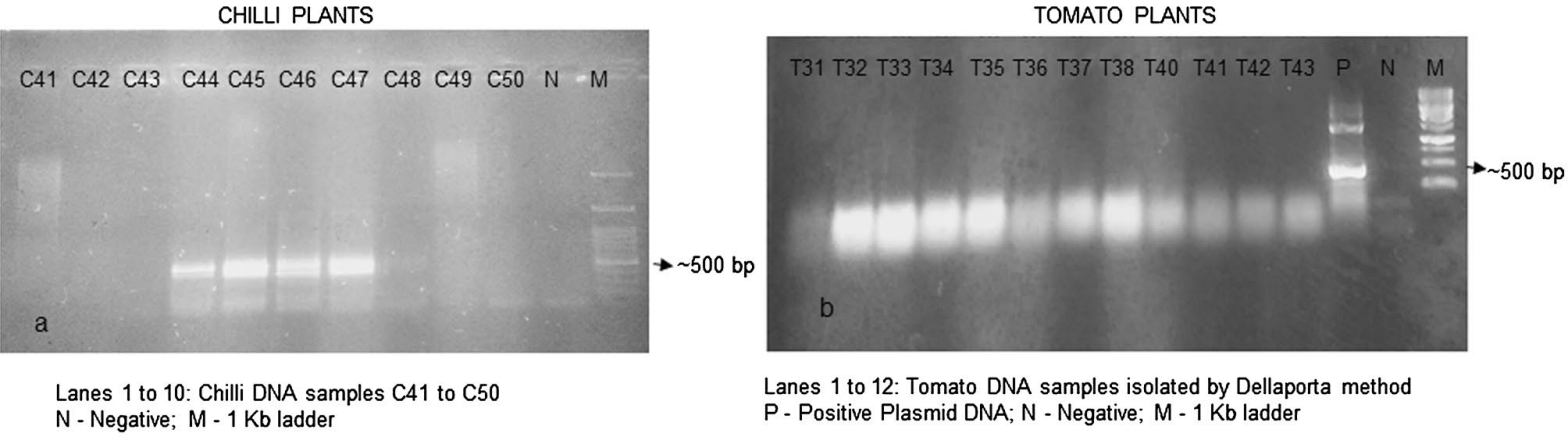
Screening of plants for the presence of begomovirus by PCR using Deng primers (Deng et al., 1994). (**a**) Lanes 1 to 10: Chilli DNA samples C41 to C50; N—Negative; M—1 Kb DNA ladder. (**b**) Lanes 1 to 12: Tomato DNA samples isolated by Dellaporta method; *P* positive plasmid DNA; *N* negative; M—1 Kb DNA ladder.

The chilli and tomato samples (99 No’s and 79 No’s) were then screened for begomovirus infection by PCR using Deng primers. PCR was also done for housekeeping gene 18S rRNA for both chilli and tomato samples and amplification was observed for chilli samples but not for tomato samples (data not shown)^54^. Out of 99 chilli DNA samples, 49 samples were found to be positive for PCR with Deng primers (amplicon of ≅ 530 bp) (Fig. 2a). PCR analysis of a few tomato samples using Deng primers were negative (Fig. 2b).

### Cloning and sequencing of coat protein region of begomovirus

**Figure 3:**
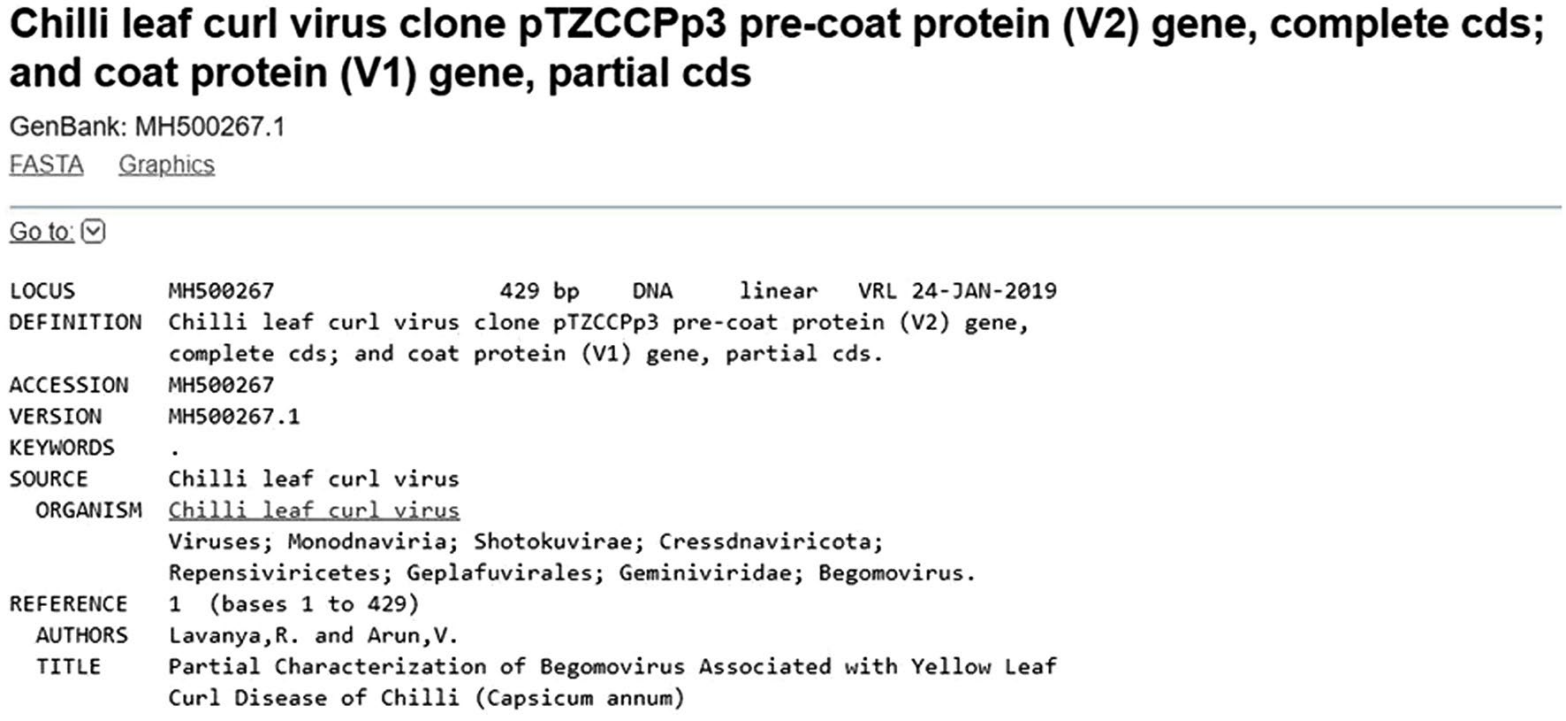
Chilli leaf curl viral coat protein gene (partial) obtained in this study.

The begomoviral DNA was amplified from chilli samples using Deng A and Deng B primers and the PCR positive chilli plants (49 samples) had an amplicon of ~ 530 bp. One of the PCR products was cloned and sequenced using M13 sequencing primers. The sequence obtained from the clone, pTZCCPp3 was then analyzed by blastn and was found to be coat protein (partial) of chilli leaf curl virus^54^. The sequence was then submitted to Genbank with the accession number MH500267 (Fig. 3).

### Synthesis and conjugation of AuNPs with clccpi1, chrppro1 and torppro1 probes

**Figure 4:**
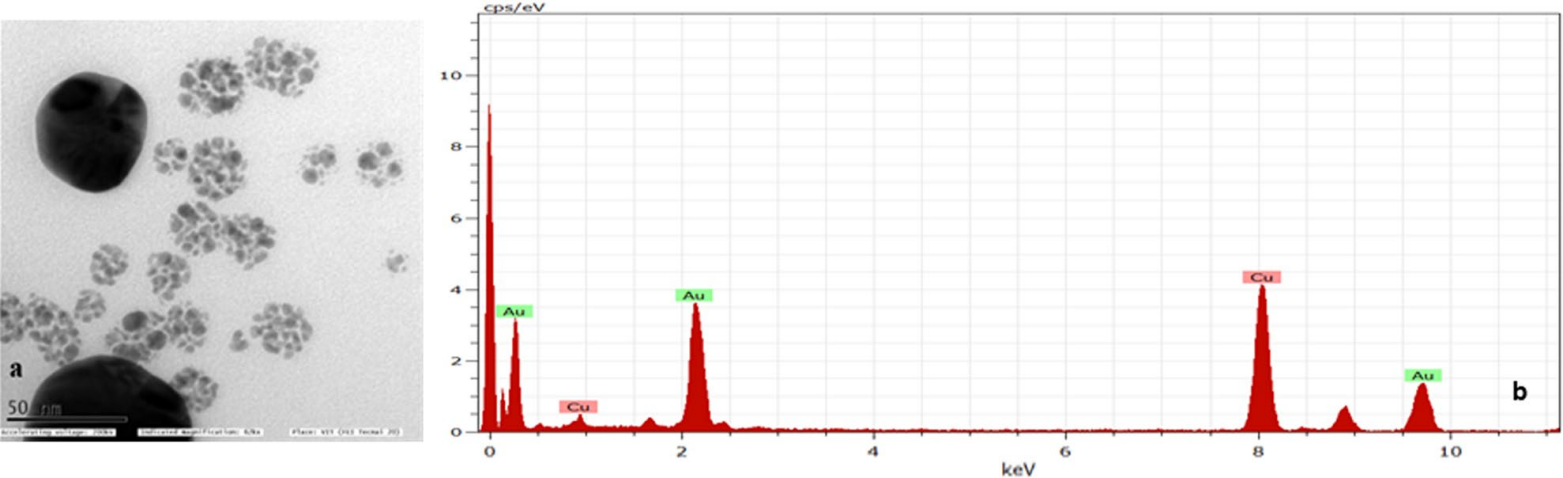
Microscopic and spectroscopic analysis of AuNPs. (**a**) Transmission Electron Micrograph (TEM) of AuNPs. (**b**) Energy Dispersive X-ray (EDX) spectroscopic analysis of synthesized AuNPs.

**Figure 5:**
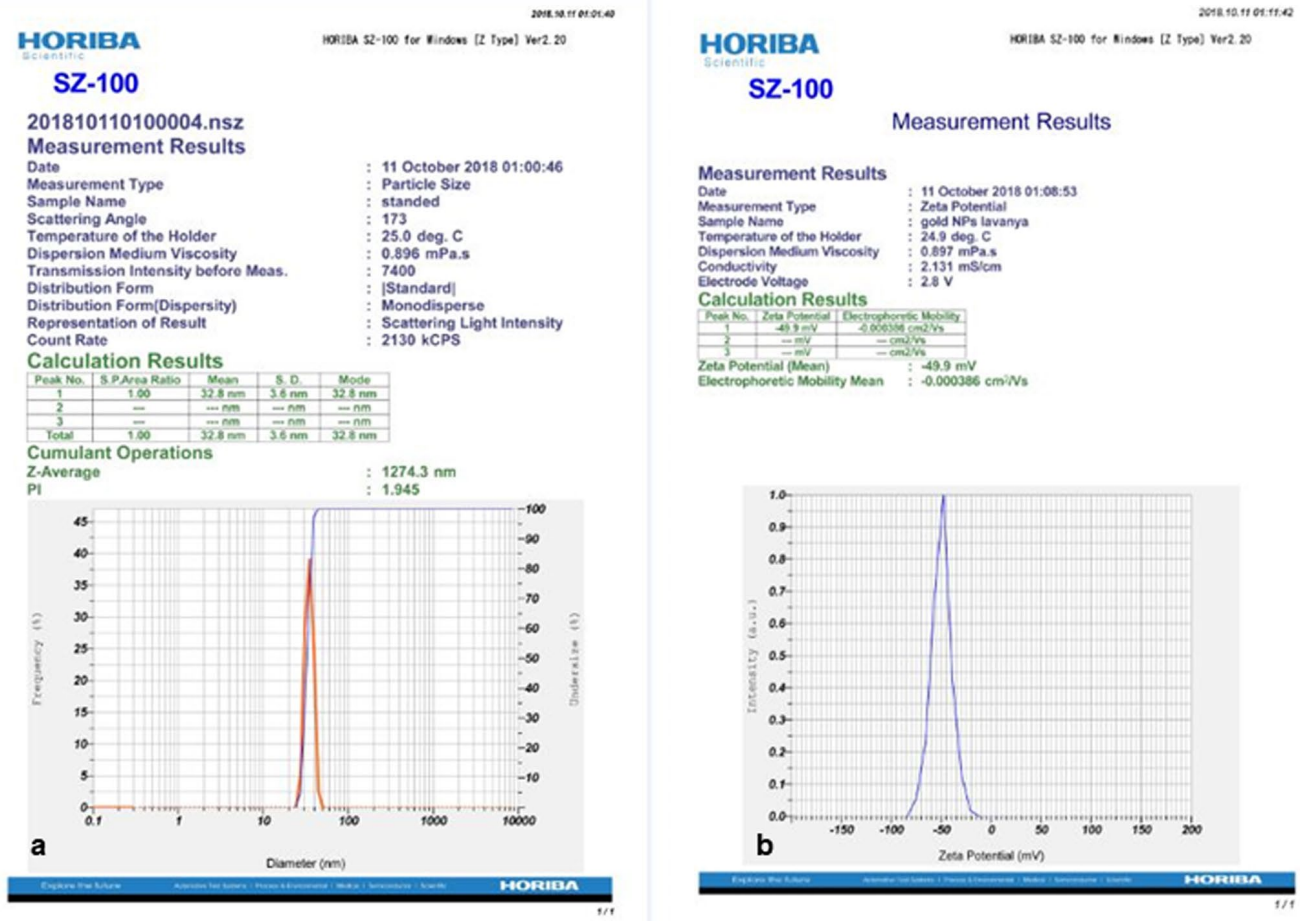
(**a**) Particle size analysis of functionalized AuNPs. (**b**) Zeta potential of functionalized AuNPs.

AuNPs synthesized by citrate reduction method were cherry red in colour and were further characterized by TEM and EDX (Fig. 4a,b). The size of the unmodified AuNPs was found to be ≅ 20 nm and the elemental composition analysis showed the presence of gold. Gold nanoparticles were then conjugated with thiolated probes (clccpi1 thiol, chrppro1 thiol and torppro1 thiol) by salt-ageing method and were found to retain the red colour. The average size of functionalized gold nanoparticles was found to be ≅ 32.8 nm and the Zeta potential of functionalized gold nanoparticles was found to be − 49.9 mV (Fig. 5a,b).

### Screening of plants for begomoviral infection by AuNP assay

**Figure 6:**
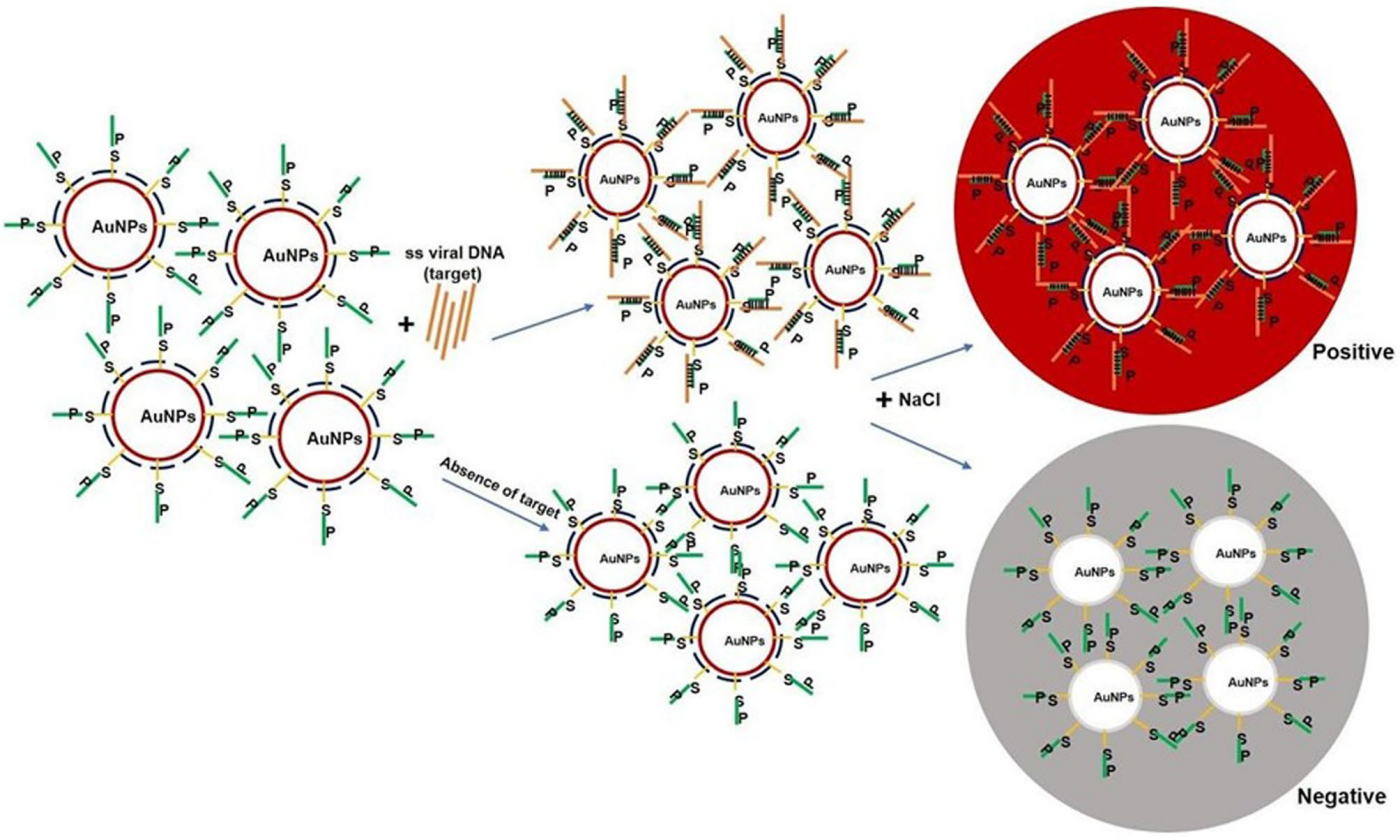
Schematic illustration of the principle of the AuNP assay for begomoviral detection. In the presence of viral DNA (target) the functionalized AuNPs retain red colour when NaCl was added as dsDNA (viral DNA + Probe DNA) prevents aggregation of the AuNPs. In the absence of viral DNA, functionalized AuNPs aggregate and turn purple due to the neutralization of electron cloud of AuNPs by the salt^55^. S-thiol linkage; P-probe.

Surface plasmon resonance property of the functionalized gold nanoparticles was utilized for the development of AuNP assay. The reaction mixture consisted of template DNA (total DNA) from plants, functionalized AuNPs and salt. When the samples retained the red colour, it indicated the presence of begomovirus whereas change of colour from red to purple indicated the absence of begomovirus. The red colour of functionalized AuNPs in the presence of viral DNA was due to prevention of aggregation of AuNPs by the dsDNA when salt was added. In the absence of viral DNA, the functionalized AuNPs (ssDNA) are freely available for interaction with salt thereby resulting in aggregation (purple colour)^55^ (Fig. 6).

### Chilli plants

**Figure 7:**
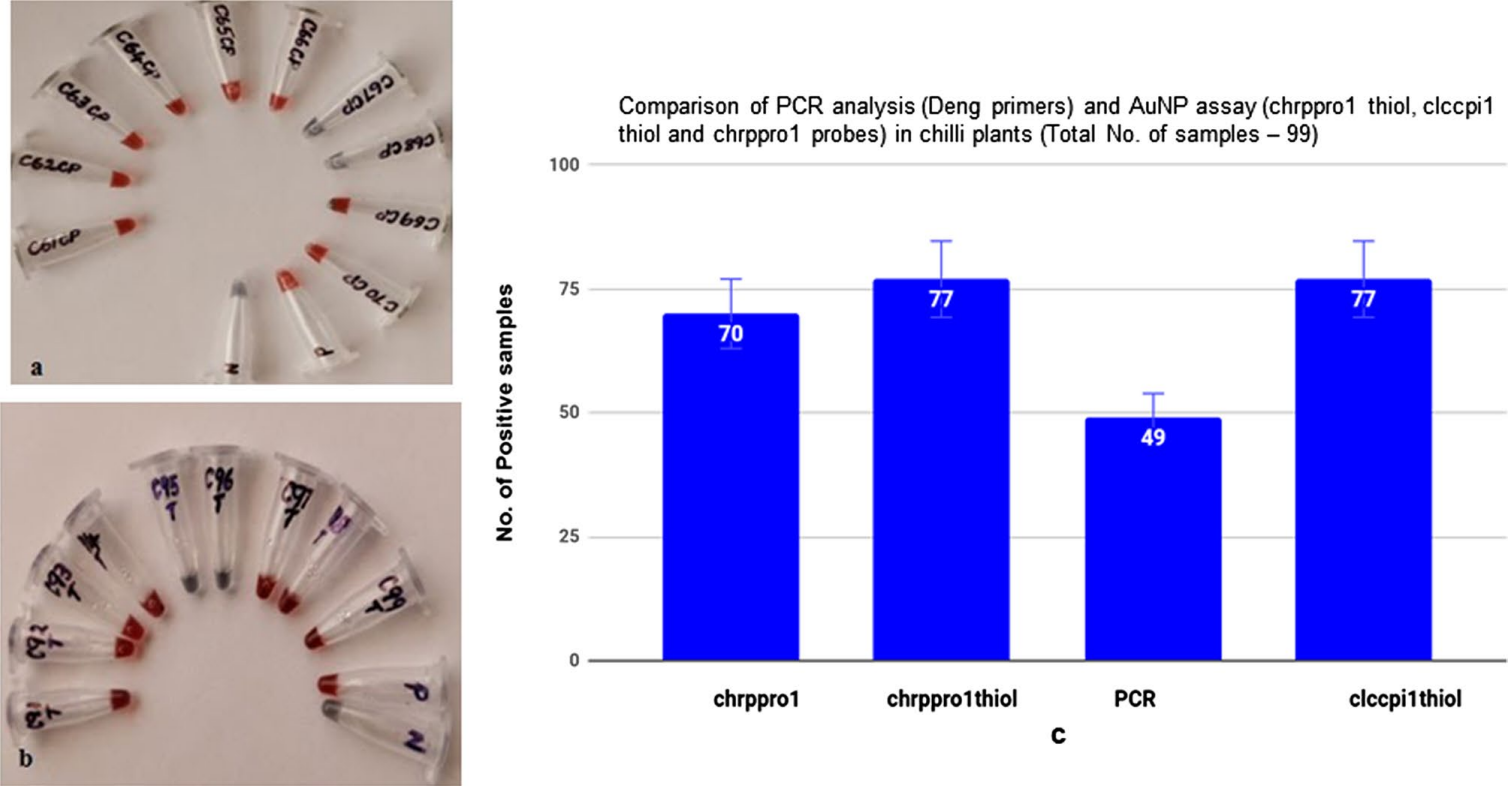
Detection of begomovirus in chilli samples using functionalized AuNPs. (**a**) With clccpi1 thiol probe; plant DNA (C71 to C80); positive (P)—begomovirus infected chilli DNA; N—healthy chilli DNA. (**b**) With chrppro1 thiol probe; plant DNA (C28 to C40); positive (P)—begomovirus infected chilli DNA; N—healthy chilli DNA. (**c**) Comparison of PCR and AuNP assay with different probes for chilli samples.

The AuNP assay was performed in duplicates for all the 99 chilli samples. AuNP assay was carried out with three sets of probes (clccpi1 thiol, chrppro1 thiol and chrppro1). Data of the AuNP assay of chilli samples with clccpi1 thiol probe and chrppro1 thiol probe was provided (Fig. 7a,b). Out of 99 samples, 77 samples were found to be positive with AuNP assay (clccpi1 thiol and chrppro1 thiol probes), whereas 70 samples were positive with chrppro1 probe (non-thiolated). PCR (Deng primers) analysis detected only 49 samples as positives (Fig. 7c).

### Tomato plants

**Figure 8:**
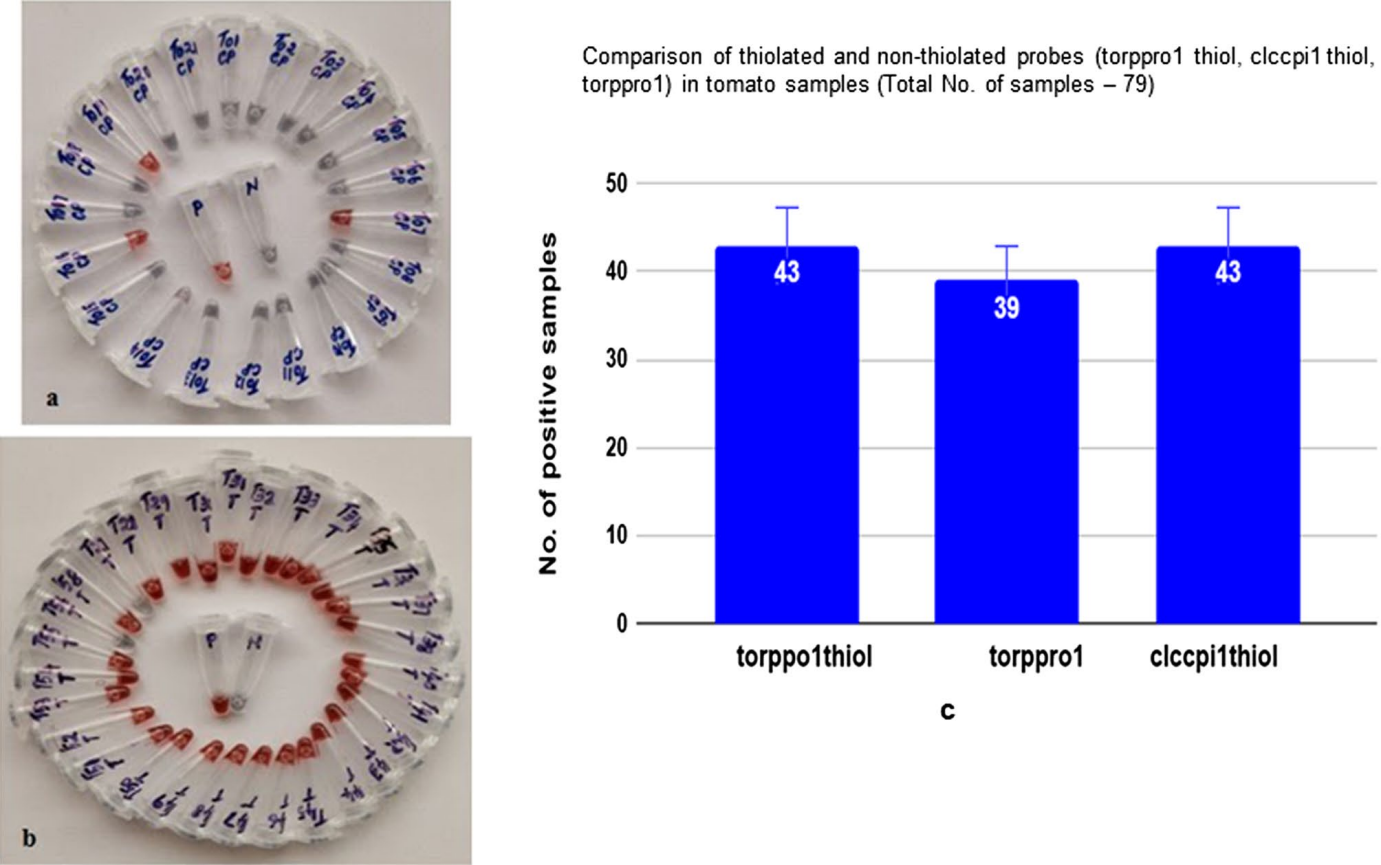
Detection of begomovirus in tomato plants using functionalized AuNPs. (**a**) With clccpi1 thiol probe, plant DNA (T27 to T38; T40 to T56 & T58), positive (P)—begomovirus infected tomato DNA, N—healthy tomato DNA. (**b**) With torppro1 thiol probe, plant DNA (To1 to To21), positive (P)—begomovirus infected tomato DNA, N—healthy tomato DNA. (**c**) Comparison of AuNP assay with different probes for tomato samples.

The AuNP assay was performed in duplicates for 79 tomato samples. The AuNP assay was carried out with three sets of probes (torppro1 thiol, torppro1 and clccpi1 thiol). Data of the AuNP assay of tomato samples with clccpi1 thiol probe and torppro1 thiol probes was provided (Fig. 8a,b). Out of 79 samples, 43 samples were found to be positive with AuNP assay (clccpi1 thiol probe and torppro1 thiol probes) and 39 samples were positive with torppro1 probe (Fig. 8c).

### Sensitivity and specificity of the AuNP assay

**Figure 9:**
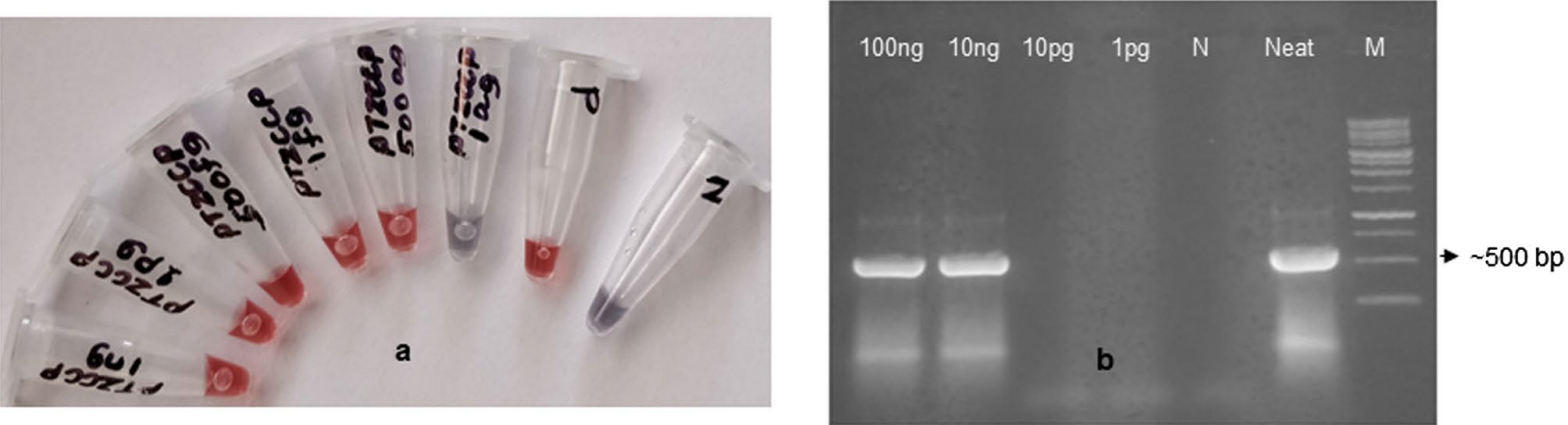
Comparison of the sensitivity of the AuNP assay and PCR (M13 primers) using different concentrations of plasmid (pTZCCPp3) diluted with Milli Q water. (**a**) AuNP assay with clccpi1 thiol probe was able to detect plasmid up to 500 ag/µl. (**b**) PCR was able to detect plasmid up to 10 ng/µl.

**Figure 10:**
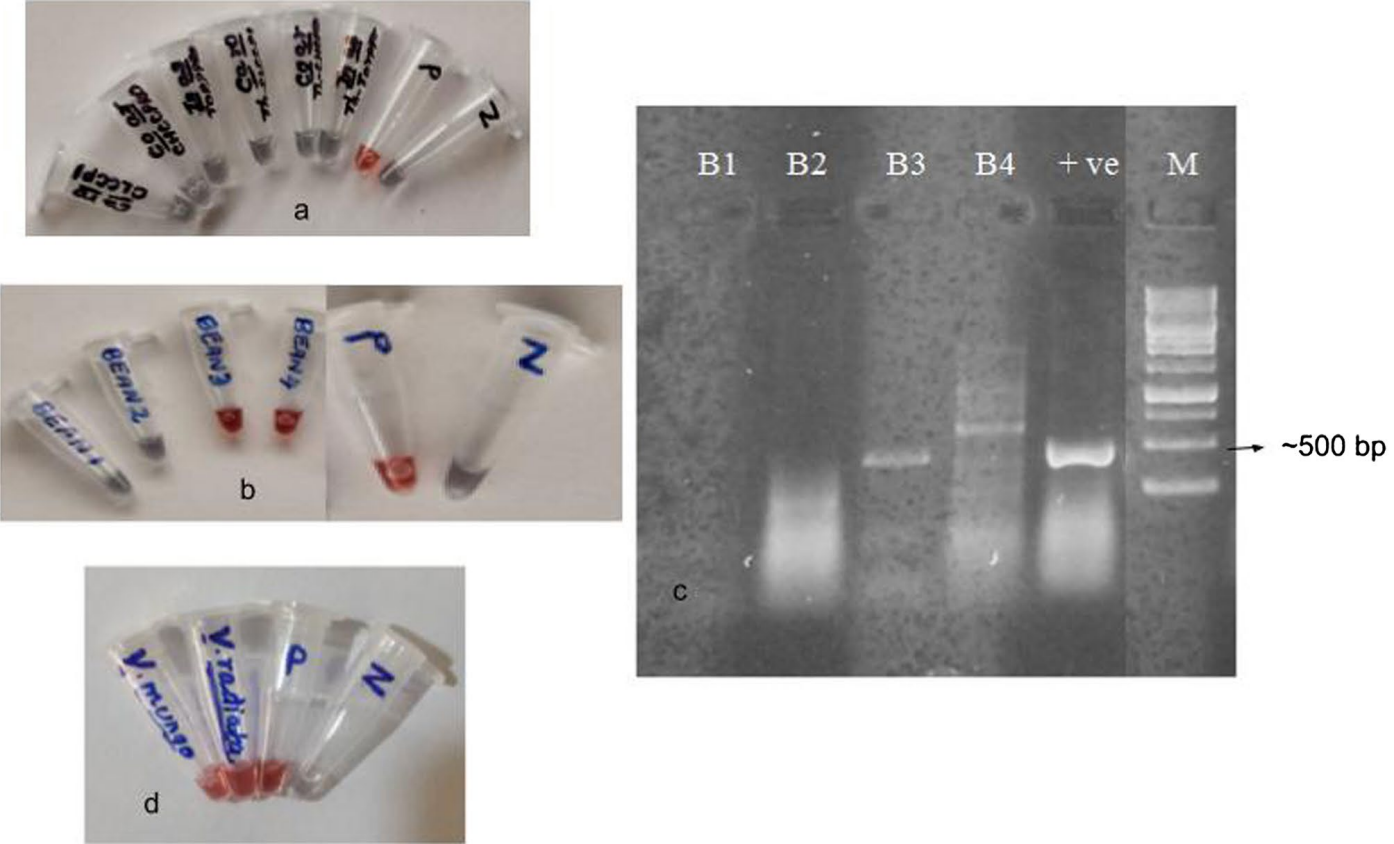
Specificity and versatility of AuNP assay for the probe—clccpi1 thiol. (**a**) Using unrelated plant DNA of three plants *I*. *aspalathoides*, *P*. *amboinicus* and *C*. *roseus*. (**b**) Using plants belonging to other family (Fabaceae)—*P. vulgaris* (common bean) that are infected by different begomoviruses. (**c**) PCR detection of begomovirus infection in bean plants (B1 to B4) using Deng primers. (**d**) Screening of begomovirus infected positive samples *V*. *mungo* (black gram) and *V*. *radiata* (mung bean) belonging to family Fabaceae were procured from TNAU. In 8c, the marker lane was from the same gel, refer supplementary figure (Supplementary file).

To test the sensitivity of the AuNP assay with clccpi1 thiol probe, plasmid (pTZCCPp3) carrying partial coat protein region of begomovirus was diluted to different concentrations (1 ng/µl to 1 ag/µl) using Milli Q water and assayed. Similar concentrations were also screened by PCR using Deng primers. The AuNP assay was able to detect plasmid up to 500 ag/µl, whereas PCR detected plasmid up to 10 ng/µl (Fig. 9a,b). The specificity of the AuNP assay with clccpi1 thiol probe was tested with total DNA from three plants belonging to different genera (*Indigofera aspalathoides*, *Plectranthus amboinicus* and *Catharanthus roseus*) that are not affected by begomovirus were chosen and AuNP assay was found to be negative (Fig. 10a).

### Versatility of the AuNP assay

Symptomatic and asymptomatic samples (4 Nos.) of common bean (*Phaseolus vulgaris*) were collected from Chennai, Tamil Nadu. Begomovirus infected plants (*Vigna mungo* and *Vigna radiata*) were obtained from Tamil Nadu Agricultural University, India (Dr. K K Kumar, Department of Plant Biotechnology) and served as positive control. The AuNP assay with clccpi1 thiol probe was performed with total DNA from plants belonging to the Fabaceae family [Common bean (*Phaseolus vulgaris* [n = 4]), mung bean (*Vigna radiata*, [n = 1]) and black gram (*Vigna mungo*, [n = 1])]. Of the 4 common bean samples, 2 were found to be positive by PCR and AuNP assay. The PCR analysis with Deng primers showed an amplicon of ~ 530 bp and ~ 600 bp for B3 and B4 bean samples respectively (Fig. 10b,c). The clccpi1 thiol probe (coat protein gene) was able to detect the Mung bean yellow mosaic virus in both Vigna varieties (Fig. 10d). The probes based on replicase gene of chilli and tomato (chrppro1 thiol and torppro1 thiol) were able to detect only the begomovirus of chilli and tomato respectively (data not shown).

## Discussion

### Screening of plants for begomovirus by PCR

The total DNA was isolated from all the plants (symptomatic and asymptomatic) under study using the Dellaporta method^56^. The total DNA isolated from the plants had a 260/280 ratio around 1.8–2 when analyzed in nanodrop and intact bands were observed on agarose gel. These confirmed that most of the genomic DNA was intact and could be used for PCR except for tomato samples which were pale brown to brown due to phenolics. PCR analysis of the plants (chilli and common bean) for 18S rRNA and begomovirus DNA (Deng primers) showed amplification whereas tomato samples were negative for PCR analysis (both primers) (Figs. 2, 10c). The absence of bands in PCR analyses of tomato plants could be due to the excessive phenolics and polysaccharides observed in tomato DNA samples, which were known to inhibit polymerase activity^57^.

Previous reports on screening of begomovirus in plants have shown utilization of different primers with varied success^58–61^. Deng primers (Deng A and Deng B) were designed on the coat protein (CP) of begomoviruses as the CP gene was highly conserved across the genus^62^. The CP gene has a significant role in encapsidation of the viral DNA and is implicated in viral movement within the plant as well as in whitefly transmission^9,63–65^. Hence for PCR detection, the most common target is the begomoviral coat protein (CP) gene^66,67^ and hence Deng primers were utilized in this study. Among the chilli plants (99 No’s) screened for begomoviral infection by PCR, 49 samples were found to be positive. The PCR with 18S rRNA for all the chilli plants were found to be positive, thus confirming the absence of bands in PCR with Deng primers was due to the absence of begomovirus and not due to the presence of any inhibitors in those samples. The coat protein region of begomovirus from a PCR positive chilli plant was amplified using Deng primer, cloned and sequenced^54^. The sequence analysis revealed the presence of a partial coat protein sequence and was deposited in GenBank with accession number (MH500267) (Fig. 3).

### Probe design

Five different probes (3 thiolated and 2 non-thiolated) based on coat protein (1 probe) and replicase protein (4 No’s) of begomovirus were designed and used in this work (Table 1). The replicase gene probes (thiolated and non-thiolated) (Table 1) for chilli and tomato plants were designed based on GenBank sequences, FM877858.1 & AY260505.1 respectively. The coat protein probe (thiolated) for chilli plant was designed based on the sequence obtained in this study (MH500267.1). Different probes (coat protein and replicase gene) were designed to determine if there are any differences in the detection limit or specificity among the probes.

**Table 1:** Probes used in this study.

Probes	Sequence (5′ → 3′)
clccpi1thiol	[ThiC6]TTCCGATTCATGGGCCTGTT
chrppro1	TTGACATCGGAGCTGGACTTTGCT
chrppro1thiol	[ThiC6]TTGACATCGGAGCTGGACTTTGCT
torppro1	ACTGCATTCTTGATTGCCCACTGC
torppro1thiol	[ThiC6]ACTGCATTCTTGATTGCCCACTGC

### AuNP synthesis and conjugation with probes

Gold nanoparticles (AuNPs) synthesized were cherry red in colour, average size of the unmodified AuNPs was found to be ~ 20 nm and elemental analysis confirmed the presence of Au in the colloidal suspension (Fig. 4a). The AuNPs were then conjugated with probes either by salt-ageing method (thiolated probes) or pH dependent citrate method (non-thiolated probes). All the functionalized AuNPs remained red in colour thus confirming the stability of AuNPs upon functionalization with different probes. Particle size analysis and Zeta potential measurement of the functionalized AuNPs with chrppro1 thiol probe were 32.8 nm and − 49.9 mV respectively, which confirmed the formation of negative charges on the surface of synthesized AuNPs. Negative charge on AuNPs is due to citrate which acts as both a reducing and a stabilizing agent that results in repulsion between AuNPs thus preventing the aggregation of AuNPs^68^. A narrow peak pattern revealed that functionalized gold nanoparticles possessed good stability (Fig. 5a,b).

### AuNP assay

AuNP assay is based on hybridization of a specific probe (coat protein or replicase gene of begomovirus) and its target DNA (begomoviral DNA). Upon hybridization, the mixture was treated with salt to detect the presence of begomovirus. Retention of red colour indicated the presence of begomovirus as the AuNPs were protected due to hybridization of probe and viral DNA (coat protein or replicase gene). In the absence of begomovirus, the probes on the AuNPs are free and upon addition of salt, the negative charges (electron cloud) on the AuNPs get neutralized thereby resulting in a change of colour from red to purple^55,69^ (Fig. 6). AuNP assay with thiolated probes (clccpi1 thiol and chrppro1 thiol) detected 77 chilli plants as infected with begomovirus and 70 samples as positive with non-thiolated probe ( chrppro1). The difference in the number of positive samples between the probes (thiolated and non-thiolated) showed that thiolated probes were better than non-thiolated probes (Fig. 7a,b). This could be due to ordered and spacious arrangement of thiolated probes onto AuNPs than non-thiolated probes, as low concentrations of thiolated probes (~ 10 nM) were used in salt-ageing method as compared with pH based citrate conjugation method (non-thiolated probe—~ 100 µM). AuNP assay was found to be better than PCR in detecting begomoviral infection in chilli plants as AuNP assay detected 77 samples as infected whereas PCR detected only 49 samples (Fig. 7c).

AuNP assay of 79 tomato samples showed that 43 samples were infected with begomovirus. Three probes (clccpi1 thiol, torppro1 thiol and torppro1) were used in the assay and the thiolated probes detected an equal number of samples (43 No’s) whereas non-thiolated probe detected 39 samples as infected (Fig. 8a–c). This showed that the AuNP assay was not affected by the presence of phenolics or polysaccharides that hamper methods like PCR due to inhibition of polymerase activity^57^. Recently, DNA templated 3D self-assembly of AuNPs clusters using bi-functional oligonucleotide probe was developed for tomato leaf curl virus^68^. Another method using LAMP PCR was developed that detected Indonesian begomovirus isolates (ToLCNDV, PepYLCIV, and TYLCKaV) simultaneously and rapidly under field conditions for routine survey^70,71^. But in this method, there is a need to employ a pre-ordered mix and a small instrument and it takes more than 30 min to complete the assay and only few samples could be assayed.

In our lab, we have developed an AuNP probe based method for the detection of BBTV in banana plants and was found to be better than PCR^53^. Overall, the detection efficiency of AuNP assay (77.7%) was found to be better than PCR screening (49.4%) in case of chilli plants. Thiolated probes (coat protein and replicase genes) were found to detect more begomoviruses than non-thiolated probes in chilli and tomato plants in AuNP assay. Among the probes, clccpi1 thiol (based on coat protein region of chilli leaf curl virus) was found to be unique as it was able to detect begomovirus of both chilli and tomato plants efficiently (Figs. 7, 8). Since the clccpi1 thiol probe was designed on the conserved region of coat protein of chilli leaf curl virus it was able to detect begomoviruses infecting chilli and tomato plants belonging to the *Solanaceae* family. The probes based on replicase genes of chilli and tomato (chrppro1 thiol and torppro1 thiol) were able to detect only begomovirus of chilli and tomato samples respectively thus confirming their specificity.

### Sensitivity and specificity of AuNP assay with clccpi1 thiol

The clone (pTZCCPp3) that carried the partial coat protein gene of begomovirus was diluted in MilliQ water and used to determine the detection limit of both AuNP assay and PCR analysis (Deng primers). AuNP assay detected plasmid upto 500 ag/µl whereas PCR detected plasmid only upto 10 ng/µl (Fig. 9). This confirmed that AuNP assay had better sensitivity than PCR and the detection limit was similar to our earlier work with BBTV^53^. The specificity assay was performed with total DNA from different healthy plants not affected by begomoviruses and was found to be negative thereby confirming that the probe (clccpi1 thiol) was specific to begomoviral DNA and not to plant DNA (Fig. 10a).

### Versatility of AuNP assay

To further validate the versatility of the clccpi1 thiol probe, plants belonging to Fabaceae family that are prone for begomoviral infection were also screened. Three samples viz*.,* common bean (*Phaseolus vulgaris* [n = 4]), mung bean (*Vigna radiata*, [n = 1]) and black gram (*Vigna mungo*, [n = 1]) were screened. The viral infected leaves of mung bean and black gram were obtained from TNAU and served as positive controls. The AuNP assay was able to detect begomoviral infection in those samples (Fig. 10b–d) and PCR analysis (Deng primers) also correlated thus confirming the versatility. These results confirmed that clccpi1 thiol probe was able to detect begomoviruses infecting plants belonging to different genera (*Solanaceae* and *Fabaceae*) in a robust manner. The begomoviruses that were potentially detected by AuNP assay (with clccpi1 thiol) in this study are Chilli leaf curl virus (CLCV), Tomato leaf curl virus (ToLCV), Mung bean Yellow Mosaic Virus (MYMV) (*Vigna radiata* and *Vigna mungo*) and Bean Golden Yellow Mosaic Virus (BGYMV) (*Phaseolus vulgaris*).

Whitefly transmitted begomoviruses cause huge losses to wide varieties of crops in India and worldwide. Pepper-infecting begomoviruses occur in India, Indonesia, Africa, Mexico, Central America, and the southern United States. Begomoviruses that infect tomato are more widely distributed and seriously impact production in tropical and subtropical regions worldwide. Monopartite begomoviruses infect peppers in Asia (e.g., Chilli leaf curl virus and Pepper leaf curl Bangladesh virus) and Africa (e.g., Pepper yellow vein Mali virus), whereas the bipartite Pepper yellow leaf curl Indonesia virus occurs in Indonesia^45,72–77^. The importance and diversity of begomoviruses infecting Capsicum species have increased over the past 5–10 years^72^. On the Indian subcontinent, in particular, leaf curl disease caused by a complex of begomoviruses and beta-satellites is considered a major constraint on pepper production^78^. Efficient viral resistant crops are still in various development stages and the simplest and effective means of controlling and managing these pathogens is the earliest possible detection and removal of infected plants from the fields. In this context, the present study gains importance as we demonstrate a versatile method that is simple to perform, does not require amplification step and is highly sensitive than other methods like PCR, ELISA that are hampered by the presence of inhibitors like phenolics, polysaccharides.

## Methods

### Plant material

The leaf samples of *Capsicum annuum* (n = 99), *Solanum lycopersicum* (n = 79) (Family: Solanaceae) and *Phaseolus vulgaris* (Family: Fabaceae) (n = 4) were collected from open fields in various regions of Tamil Nadu, India. The leaf sample from the following plants, *Plectranthus amboinicus* (Family: Lamiaceae) *Catharanthus roseus* (Family: Apocynaceae) were collected from Chennai, Tamil Nadu and *Indigofera aspalathoides* (Family: Fabaceae) was collected from Madurai, Tamil Nadu. The above three plants served as internal controls (plant DNA) to determine the specificity of the probes employed. For positive control, begomoviral infected leaf samples of plants belonging to the Fabaceae family (*Vigna mungo* [n = 1] and *Vigna radiata* [n = 1]) were obtained from TNAU (Dr. K.K. Kumar, Associate Professor, Department of Plant Biotechnology, Tamil Nadu Agricultural University (TNAU), Coimbatore).

### Statement on guidelines

Experimental research and field studies on cultivated plants comply with relevant institutional, national, and international guidelines and legislation.

### Isolation of total DNA from plants

The total DNA was isolated from the plants by Dellaporta DNA isolation method^56^ with few modifications. Briefly, 2 g of leaf tissue was ground to a fine powder using liquid nitrogen, transferred to a sterile falcon tube (50 ml) followed by the addition of 5 ml of extraction buffer [100 mM Tris (pH 8.0), 50 mM EDTA, 500 mM NaCl, and 10 mM β-mercaptoethanol]. The mixture was vortexed and 660 µl of 10% SDS was added, mixed thoroughly and incubated at 65 °C for 10 min. Then, 1.6 ml of 5 M potassium acetate was added and centrifuged at 8000 rpm for 15 min at 4 °C. The supernatant was transferred to a fresh tube and an equal volume of ice-cold chloroform was added and centrifuged at 8000 rpm for 15 min at 4 °C. The supernatant was transferred to a fresh tube and an equal volume of ice-cold isopropanol was added, mixed and allowed to rest for 15–20 min at room temperature. The total DNA was precipitated by centrifugation at 8000 rpm for 15 min at 4 °C. The pellet was washed with 70% ice-cold ethanol at 8000 rpm for 10 min. The pellet was air-dried and resuspended in 30–50 µl of sterile distilled water. The quality and quantity of the total DNA were analyzed using Nanodrop.

### Screening of begomovirus in plants by PCR

Total DNA isolated from plants (chilli—99 No’s, tomato—79 No’s, common bean—4 No’s) were screened for begomovirus using Taq DNA Polymerase 2X Master Mix RED with 1.5 mM MgCl_2_ (Synergy Scientific) and gemini virus specific primers—Deng primers (Deng A 5’ TAATATTACCKGWKGVCCSC 3’ and Deng B 5’ TGGACYTTRCAWGGBCCTTCACA 3’)^79^ for 30 cycles and results were documented.

### Cloning and sequencing of partial coat protein of begomovirus

The PCR product (Deng primers) of one of the positive samples was cloned using InsTAclone kit and *E*. *coli* DH5α was transformed as per the instructor’s manual and one of the clones was sequenced^54^.

### Designing of probe for begomovirus detection

The probe for coat protein gene of chilli was designed based on the sequence obtained in this study and the probes for replicase gene of chilli and tomato were designed based on the GenBank sequences (FM877858.1 and AY260505.1) respectively using online tool PRIMER QUEST. Both thiolated and non-thiolated probes were obtained from Sigma Aldrich, Chennai and suspended in MilliQ water at 100 µM concentration.

### Functionalization of thiolated probes

The thiolated probes (10 nM) were functionalized by treating with freshly prepared dithiothreitol (DTT) and purified as described in Kumar et al.^53^. Briefly, 1 ml of 10 nM thiolated probe was treated with 100 µl of DTT and incubated for 30 min at RT and purified using NAP25 column as per manufacturer’s instructions (GE Lifesciences) and used for conjugation of gold nanoparticles (AuNPs).

### AuNP synthesis and conjugation with probes

Gold nanoparticles (AuNPs) were synthesized by citrate reduction method and conjugated with thiolated probes as described in earlier reports^53^ and stored at 4 °C. One gram of gold chloride [HAuCl_4_ × H_2_0 (SRL, India, Cat. No. 12023)] was suspended in 1 ml of MilliQ water and used for the synthesis of gold nanoparticles. Briefly, 50 ml of Milli Q water and 5 µl of gold chloride were mixed in a spoutless beaker and a round-bottomed flask filled with water was kept on the top to prevent evaporation. The mixture was brought to boiling temperature and 750 µl of 1% trisodium citrate dihydrate (Merck) was added and boiled further for 10 min. The mixture turned blue, then gradually to cherry red by the end of 10 min. The AuNPs thus synthesised were allowed to cool at RT and were stored in 50 ml falcon tube at 4 °C until further use.

The freshly synthesized AuNPs were conjugated with non-thiolated probes by pH dependent citrate conjugation method^80^ with slight modifications. To 1 ml of AuNPs, 5 µl of non-thiolated probe (~ 100 µM) was added, vortexed gently and 20 µl of 500 mM sodium citrate-HCl (pH 3) was added and incubated at room temperature for 3 min. Then, 60 µl of 10 mM HEPES (pH 7.6) was added, mixed by inversion and incubated at room temperature for 5 min. The mixture was then centrifuged at 12,000 rpm for 5 to 10 min. The pellet was suspended in 50 µl of resuspension buffer [10 mM phosphate buffered saline (PBS) pH 7.4; 0.1% SDS], covered in aluminium foil and stored at RT until use.

The freshly synthesized AuNPs were conjugated with activated thiolated probes by salt-ageing method^81^ with slight modifications. Briefly, to 15 ml of AuNPs, 300 µl of activated thiolated probe was added and incubated for 30 min in an orbital shaker at RT overnight for 90 rpm. Then, 297 µl of 100 mM phosphate buffer (pH 7) was added, followed by addition of 165 µl of 10% SDS. The mixture was further incubated for 30 min in an orbital shaker 90 rpm at RT. Finally, six doses of 81 µl of 2 M NaCl in 100 mM PBS (pH 7) were added to the mixture over 2 days in an orbital shaker 90 rpm at RT. The functionalized AuNPs were purified in aliquots of 1 ml in 1.5 ml centrifuge tubes by centrifugation at 12,000 rpm for 10 min and washed with 500 µl of resuspension buffer [100 mM phosphate buffered saline (PBS) pH 7.4; 0.1% SDS]. Finally, the functionalized AuNPs were suspended in 50 µl of resuspension buffer, covered in aluminium foil and stored at RT until use.

### Characterization of AuNPs

The synthesized unmodified AuNPs were characterized by Surface Electron Microscopy (SEM), Transmission Electron Microscopy (TEM) and Energy Dispersive X-ray Spectroscopy (EDX) at Sophisticated Instrument Facility (SIF), Vellore Institute of Technology (VIT). The size and zeta potential of the functionalized AuNPs were analyzed using Particle Size Analyzer (Horiba Scientific SZ-100) at TRPVB, Madhavaram, Chennai, India.

### AuNP assay for begomovirus detection

The total DNA from chilli plants (100 ± 10 ng/µl) were diluted (1:5), tomato plants (100 ± 10 ng/µl) were diluted (1:10) with MilliQ water and utilized for AuNP assay. The assay was performed in a PCR machine (G Storm) and had the following steps—diluted total DNA (5 µl) was denatured at 95 °C for 5 min followed by addition of 5 µl of functionalized AuNP and annealing was done at 65 °C for 10 min. Finally, 5 µl of 2 M NaCl prepared in MilliQ water was added and the results were recorded within 20 min. The DNA of begomovirus infected plants (chilli and tomato) served as positive control and healthy plant DNA (chilli and tomato) served as negative control and the principle of the assay was represented (Fig. 6)^55^.

### Specificity and sensitivity of AuNP assay

To confirm the specificity and sensitivity of the assay, the plasmid (pTZCCPp3 carrying partial coat protein DNA) served as positive control and MilliQ as a negative control. The total DNA from other plants belonging to the other genera, which are not susceptible to begomovirus, *Indigofera aspalathoides*, *Plectranthus amboinicus* and *Catharanthus roseus* were tested against the functionalized AuNPs. The clone (pTZCCPp3) was diluted with MilliQ water, the different concentrations (100 ng/µl, 10 ng/µl, 10 pg/µl and 1 pg/µl) were analyzed by PCR using M13 primers. Different concentrations of the clone (pTZCCPp3) (1 ng/µl, 1 pg/µl, 500 fg/µl, 1 fg/µl, 500 ag/µl and 1 ag/µl) were tested using AuNP assay.

### Ethics approval and consent to participate

Institutional ethical clearance was obtained for this study, IEC-NI/14/DEC/44/75, dated December 22, 2014. Consent to participate is not applicable.

### Consent for publication

The authors declare their consent for publication.

## Supplementary Information

Supplementary Information.

## Data Availability

The data and the materials used in the current study are available with the corresponding author and can be produced on need.
