# Supplementary material for: Detection of Begomovirus in chilli and tomato plants using functionalized gold nanoparticles
Source: Sci Rep. 2021 Jul 9;11:14203. doi: 10.1038/s41598-021-93615-9 (PMC8271019; doi:10.1038/s41598-021-93615-9)
Supplement: Supplementary file 1 — Supplementary Information. [file 41598_2021_93615_MOESM1_ESM.doc]

**Detection of Begomovirus in chilli and tomato plants using functionalized gold nanoparticles**

**Affiliation:**

**Department of Biotechnology, Sri Ramachandra Institute of Higher Education and Research, Chennai, Tamil Nadu, India.**

Lavanya R^1^ and Arun V*^, 1^

Supplementary Figure

**Fig 8c**


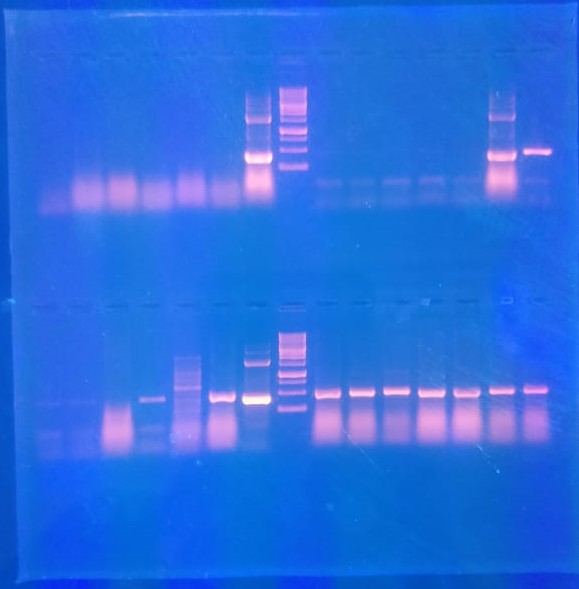


**B1 B2 B3 B4 +ve +ve**

**B1**

**M**

**Fig. 8** Specificity and versatility of AuNP assay for the probe - clccpi1 thiol

**8c** PCR detection of begomovirus infection in bean plants (B1 to B4) using Deng primers **d** Screening of begomovirus infected positive samples *V*. *mungo* (black gram) and *V*. *radiata* (mung bean) belonging to family Fabaceae were procured from TNAU

**In Fig 8c of manuscript, few lanes were cropped to provide the data from the above gel.**

**The top lanes were PCR products (Deng primers) of tomato and papaya plants and most of the samples were negative and hence avoided.**

**In the bottom lanes, sample B1 was loaded twice, hence one of the lanes was not included in the manuscript. The immediate lane (*) next to +ve lane represent PCR product using higher concentration of the clone, pTZCCPp3 and hence it was not included in the manuscript. The lanes after the maker, M represent PCR products obtained from a different study and hence not included in the study.**
